# Mitogen-Activated Protein Kinase Kinase 4 Gene Polymorphism and Cancer Risk

**DOI:** 10.1097/MD.0000000000000938

**Published:** 2015-11-06

**Authors:** Peiliang Geng, Juanjuan Ou, Ganfeng Xie, Jianjun Li, Xiaoxin Zhao, Lisha Xiang, Yunmei Liao, Ning Wang, Houjie Liang

**Affiliations:** From the Department of Oncology and Southwest Cancer Center, Southwest Hospital, Third Military Medical University, Chongqing, China.

## Abstract

A number of epidemiological studies have assessed the association of −1304T > G polymorphism in the *MKK4* gene and risk of cancer, but the results lack of statistical power due to the limited subjects used in these studies. This study was devised to identify the genetic effects of the −1304T > G polymorphism on cancer risk in a large meta-analysis.

Eligible studies were identified by searching both Chinese and English databases. General as well as subgroup analyses were performed for 8 independent case–control publications with a total of 4623 cases and 5256 cancer-free controls. Odds ratios (ORs) and 95% confidence intervals (CIs) were used to estimate the association.

Overall, this meta-analysis showed that the association between the −1304T > G polymorphism and cancer risk was statistically significant (GG vs TT: OR = 0.63, 95% CI, 0.52–0.75; GG + TG vs TT: OR = 0.85, 95% CI, 0.79–0.91; GG vs TG + TT: OR = 0.67, 95% CI, 0.56–0.80; G vs T: OR = 0.82, 95% CI, 0.77–0.88; TG vs TT: OR = 0.86, 95% CI, 0.79–0.93).

Our meta-analysis reveals that the presence of the −1304T > G polymorphism is likely to decrease risk of cancer. Future larger studies are necessary to validate the current finding.

## INTRODUCTION

Environmental carcinogens interacting with inherited factors is the result of cancer.^1,2^ The interaction is directly or indirectly involved in the activation of the mitogen-activated protein (MAP) kinase pathways that converge on c-Jun N-terminal kinases (JNKs) and p38 MAPKs and function as essential regulators of cellular senescence.^3^ Mitogen-activated protein kinase 4 (*MKK4*, also known as MAP2K4, MEK4, JNKK1, and SEK1) is a member of the MAP kinase family, playing a key role in multiple physiologic and pathophysiologic processes such as inflammation and tumor suppression.^4^*MKK4* is highly mutated and has a pro-oncogenic role in cancers of pancreatic, breast, colon, prostate,^5^ skin,^6^ and laryngeal squamous cell.^7^ Meanwhile, *MKK4* has also been proved as a suppressor gene in the metastasis of prostate and ovarian cancer cell lines.^8,9^ Its function in tumorigenesis remains highly controversial.

Mapped to chromosome 17p11.2, the *MKK4* gene encoding a 399-amino acid protein in humans spans over 120 kb and consists of 11 exons.^4,10^ A previous study reporting the association between −1304T > G (rs3826392) polymorphism in the promoter of *MKK4* gene and the risk of sporadic colorectal cancer in a southern Chinese population showed a decreased risk correlated with the −1304T > G polymorphism.^11^ Identical results were also reported in a follow-up investigation focusing on colon caner.^12^ A number of genetic epidemiological studies looking at other cancers presented substantial evidence that the functional role of the −1304T > G polymorphism in cancer risk differs depending on the type of cancer.^13–18^ Since the findings are on the ground of relatively small samples restricted to a specific population, thus they may have been underestimated and lack statistical power to elucidate the underlying mechanism of cancer onset associated with this polymorphism.

In an effort to identify the genetic effects of the −1304T > G polymorphism on cancer risk, we performed a meta-analysis composed of the publications evaluating the association between the −1304T > G polymorphism and risk of cancer.

## METHODS

### Identification and Eligibility of Relevant Studies

We searched both English (PubMed, Embase) and Chinese databases (CNKI) for all publications regarding the association between the −1304T > G polymorphism and cancer risk by using the keywords: *MKK4*, −1304T > G/rs3826392, polymorphism/polymorphisms/variant/genotype/SNP, and cancer. All references cited in the retrieved articles were also screened to identify the missing data eligible for the meta-analysis. Publications were included in the meta-analysis when satisfying the following criteria: (a) investigating the association between the −1304T > G polymorphism and cancer risk in patients with cancer and control subjects; (b) having genotype data in full detail; (c) published as a full text, rather than a short summary or a comment letter before January 2014. The study was approved by the ethics committee of southwest hospital.

### Data Extraction

Based on the inclusion criteria and a consensus reached on all items, 2 investigators independently extracted data including first author, year of publication, country, ethnicity, control source, sex proportion in cases and controls, cancer type, genotyping method, and genotype distributions in cases and controls. In addition, we merged colon cancer into the colorectal cancer when performing meta-analysis.

### Statistical Analysis

STATA software, version 12.0 (Stata Corporation, College Station, TX) was used to analyze the data of the −1304T > G polymorphism. The fixed effects model or the random effects model was performed to calculate the odds ratios (ORs) and 95% confidence intervals (CIs) for each study. Heterogeneity between studies was measured by Chi-square-based *Q* test^19^ and *I*^2^ statistic.^20^ When no obvious inconsistency existed across the studies (*P* > 0.10 or *I*^2^ < 50%), the fixed-effect model (Mantel–Haenszel) was used to pool the ORs; otherwise, the random effects model (DerSimonian and Laird) was selected.^21,22^ Sensitivity analysis was performed by sequentially excluding each study and rechecking whether the corresponding ORs were altered significantly. Funnel plots and Egger's test^23^ were used to determine the potential publication bias in the meta-analysis. All tests were 2-sided with *P* < 0.10 being statistically significant.

## RESULTS

### Study Characteristics

**FIGURE 1:**
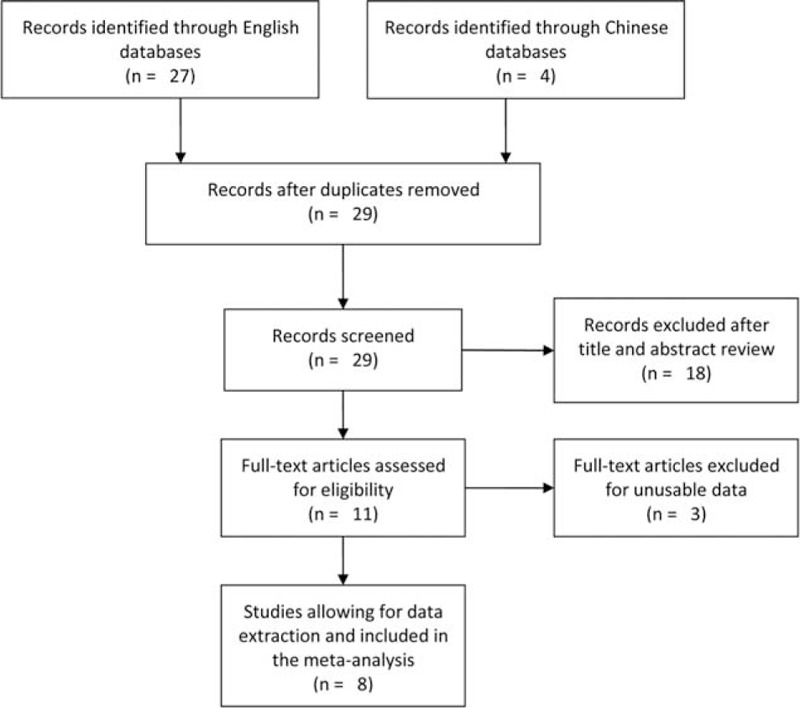
Flow diagram of the study selection process.

Through the comprehensive search, we yielded a total of 31 papers, whose eligibility was examined by reviewing the key words, titles, abstracts, and the full texts according to the predescribed criteria for inclusion. After excluding the unavailable publications, 8 studies with 4623 cases and 5256 cancer-free controls were included in this meta-analysis^11–18^ (Figure 1). Of these studies, 2 were published in Chinese^12,15^ and 6 were in English.^11,13,14,16–18^ All of the studies were conducted for the Asian populations. Table 1 lists the main characteristics of the eligible studies for this meta-analysis.

**TABLE 1:**
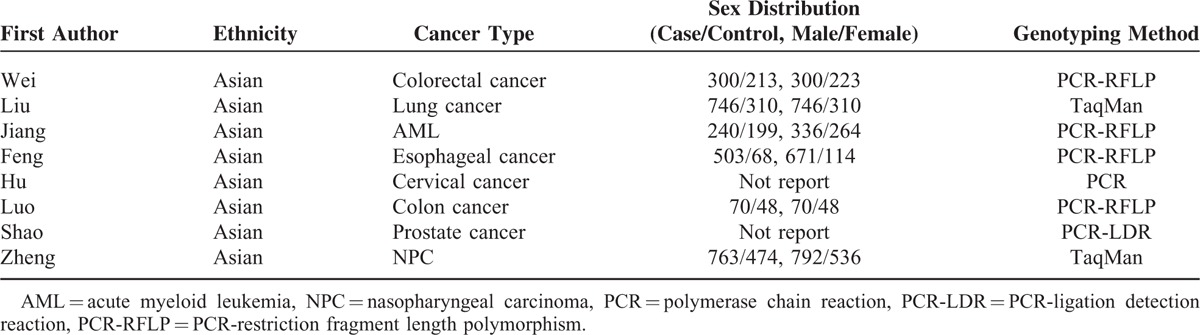
Distribution of *MKK4* −1304T>G Gene in Cancer Cases and Control Subjects

### Quantitative Synthesis

**FIGURE 2:**
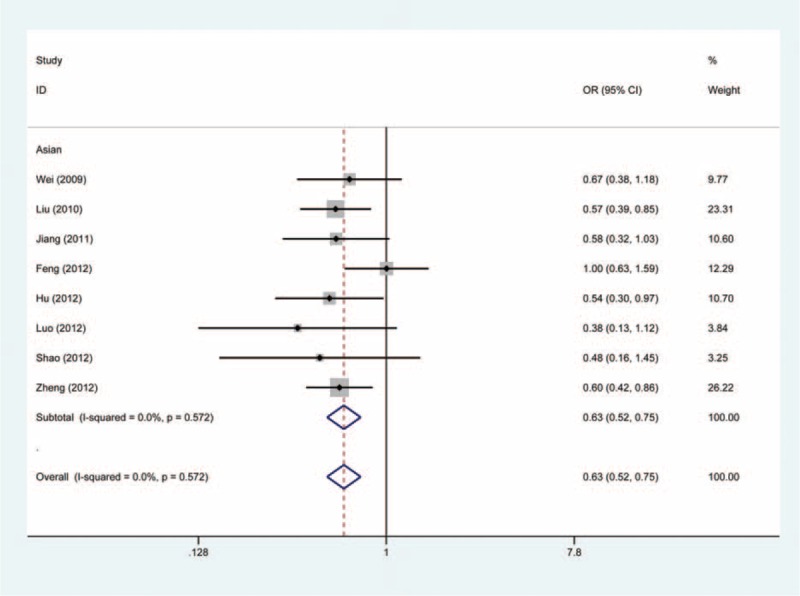
Forest plot of cancer susceptibility associated with *MKK4* −1304T>G polymorphism under GG vs TT with fixed-effects model.

**FIGURE 3:**
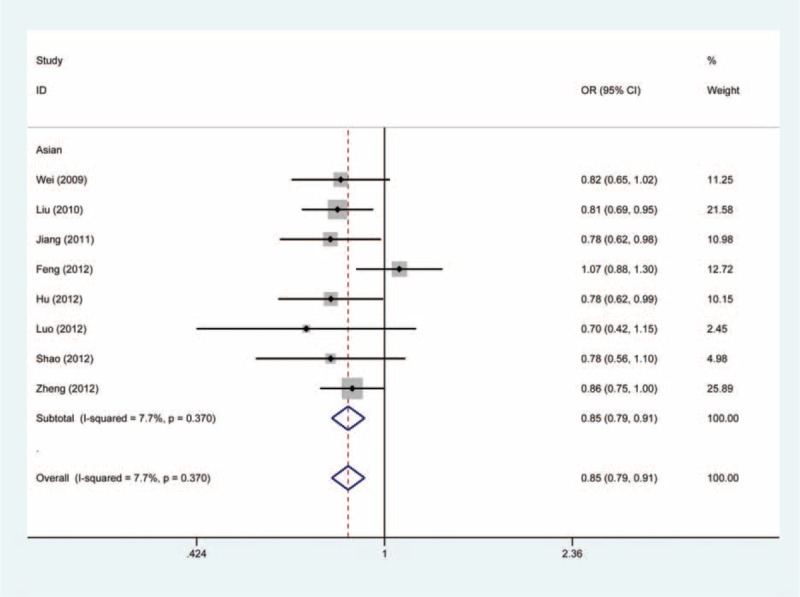
Forest plot of cancer susceptibility associated with *MKK4* −1304T>G polymorphism under GG + TG vs TT with fixed-effects model.

As shown in Table 2, pooling all data on the association between the −1304T > G polymorphism and cancer risk into a large dataset revealed a significantly reduced risk of cancer in all genetic models (GG vs TT: OR = 0.63, 95% CI, 0.52–0.75, Figure 2; GG + TG vs TT: OR = 0.85, 95% CI, 0.79–0.91, Figure 3; GG vs TG + TT: OR = 0.67, 95% CI, 0.56–0.80; G vs T: OR = 0.82, 95% CI, 0.77–0.88; TG vs TT: OR = 0.86, 95% CI, 0.79–0.93).

**TABLE 2:**
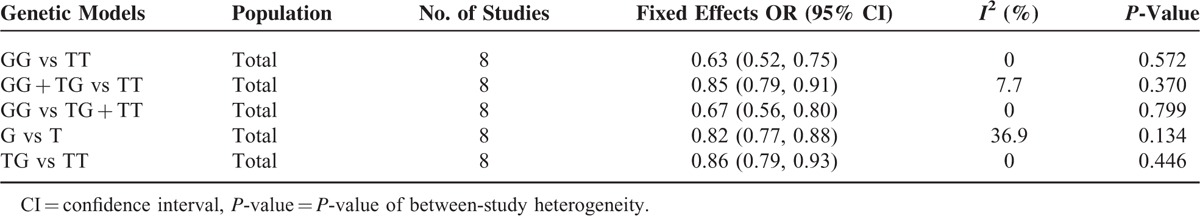
Odds Ratio (OR) and Heterogeneity Results for *MKK4* −1304T>G Gene in Various Cancers

**FIGURE 4:**
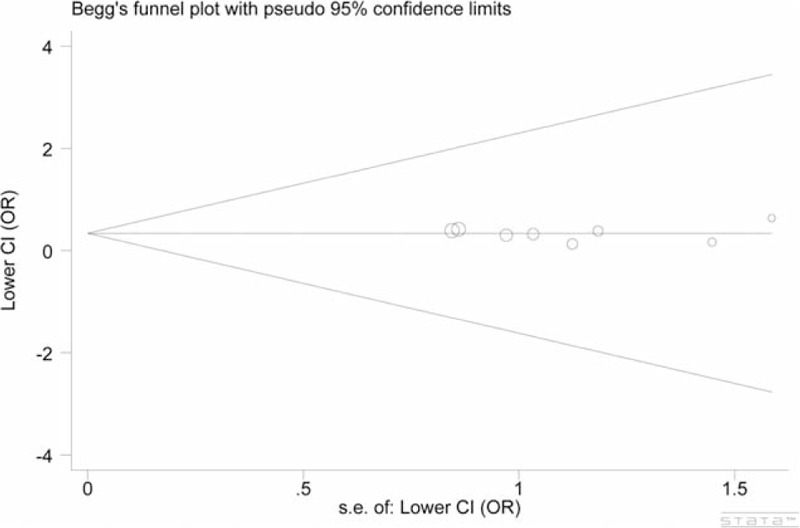
Funnel plots of *MKK4* −1304T>G polymorphism cancer risk. Model: GG vs TT, Egger's test: *P* = 0.894.

No significant between-study heterogeneity was suggested in the meta-analysis (Table 2). Further sensitivity analysis did not reveal any quantitative alternation occurring in the ORs (data not shown). In addition, the funnel plots were symmetrical (*P* = 0.902) and Egger's test suggested no evidence of obvious publication bias among the studies (GG vs TT: *P* = 0.894) (Figure 4).

## DISCUSSION

Based on 8 independent case–control publications with a total of 4623 cases and 5256 cancer-free controls, we performed a meta-analysis to comprehensively assess the relationship between the −1304T > G polymorphism and risk of different types of cancers. From the analysis results, we found statistically significant evidence for a reduction in cancer risk when combining all data together. Since the insufficient data on each cancer did not allow further stratified analysis, such as by type of cancer, thus we failed to estimate the associations of the studied polymorphism and various cancers.

*MKK4* belongs to MAPK pathways known to have involvement in the regulation of apoptosis, inflammation, and tumorigenesis.^11^ An increasing body of evidence has found that the molecular activity of *MKK4* is associated with the formation and initiation of cancers.^24–26^ Frequent mutations of *MKK4* have been reported in lung cancer and colorectal cancer.^27,28^ Also, loss-of-function mutations in the *MKK4* gene is shown in a portion of numerous human tissues, accounting for approximately 5%.^24,29^ Four common polymorphisms in the promotor region of the *MKK4* gene have been recorded in Genebank dbSNP database. The genetic variations in the promoter region could affect transcriptional activity and biological function of this gene^14^ resulting in tumorigeneses.

Since the discovery of a decreased risk associated with the −1304T > G polymorphism in the promotor region of *MKK4* was claimed in colorectal cancer,^11^ a large number of replication studies on various cancers have been successively done in recent years. Most of the studies concluded that the −1304T > G polymorphism have protective effects on the development of cancer.^12,13,16^ Nevertheless, the susceptibility to esophageal cancer was not found to be associated with this polymorphism.^15^ Although our meta-analysis revealed an decreased risk in overall cancer, we cannot rule the possibility that the functional −1304T > G polymorphism decreases risk of some cancers by increasing the promoter activity, while it has no biological significance in other cancers, such as esophageal cancer.

Several potential factors must be concerned when interpreting the findings in this meta-analysis. First, the connections of various cancer risks associated with the −1304T > G polymorphism are on the basis of small samples, possibly leading to an underestimate of the true association, which should be further confirmed in a much larger study. Second, we only searched studies conducted among Asian subjects, thus the role of the studied polymorphism in cancer should be widely investigated in more ethnic populations. Third, cancer is known to be a multifactorial disease caused by complex interactions between environmental and genetic factors.^30^ However, the estimate of such effects was not considered in the present meta-analysis, because the limited data did not allow us to do so.

In summary, this meta-analysis provides evidence that the −1304T > G polymorphism is strongly associated with a decreased risk of cancer. A large well-designed study in diverse ethnic populations is warranted to clarify the true association between the −1304T > G polymorphism and cancer.
